# Urethral injection of dedifferentiated fat cells ameliorates sphincter damage and voiding dysfunction in a rat model of persistence stress urinary incontinence

**DOI:** 10.1007/s11255-021-03083-3

**Published:** 2022-02-17

**Authors:** Yasutaka Murata, Daisuke Obinata, Taro Matsumoto, Yuichiro Ikado, Koichiro Kano, Noboru Fukuda, Kenya Yamaguchi, Satoru Takahashi

**Affiliations:** 1 Department of Urology, Nihon University School of Medicine, Tokyo, Japan; 2 Division of Cell Regeneration and Transplantation, Department of Functional Morphology, Nihon University School of Medicine, 30-1 Oyaguchi-Kamicho, Itabashi-ku, Tokyo, 173-8610 Japan; 3 Laboratory of Cell and Tissue Biology, College of Bioresource Science, Nihon University, Fujisawa, Japan

**Keywords:** Adipocyte, Dedifferentiated fat cell, Cell therapy, Vaginal distension, Ovariectomy

## Abstract

**Purpose:**

Dedifferentiated fat (DFAT) cells are mature adipocyte-derived multipotent cells that can be applicable to cell-based therapy for stress urinary incontinence (SUI). This study developed a persistence SUI model that allows long-term evaluation using a combination of vaginal distention (VD) and bilateral ovariectomy (OVX) in rats. Then, the therapeutic effects of DFAT cell transplantation in the persistence SUI model was examined.

**Methods:**

In total, 48 Sprague–Dawley rats were divided into four groups and underwent VD (VD group), bilateral OVX (OVX group), VD and bilateral OVX (VD + OVX group), or sham operation (Control group). At 2, 4, and 6 weeks after injury, leak point pressure (LPP) and histological changes of the urethral sphincter were evaluated. Next, 14 rats undergoing VD and bilateral OVX were divided into two groups and administered urethral injection of DFAT cells (DFAT group) or fibroblasts (Fibroblast group). At 6 weeks after the injection, LPP and histology of the urethral sphincter were evaluated.

**Results:**

The VD + OVX group retained a decrease in LPP with sphincter muscle atrophy at least until 6 weeks after injury. The LPP and urethral sphincter muscle atrophy in the DFAT group recovered better than those in the fibroblast group.

**Conclusions:**

The persistence SUI model was created by a combination of VD and bilateral OVX in rats. Urethral injection of DFAT cells inhibited sphincter muscle atrophy and improved LPP in the persistence SUI model. These findings suggest that the DFAT cells may be an attractive cell source for cell-based therapy to treat SUI.

## Introduction

Stress urinary incontinence (SUI) is a major health problem defined as the involuntary leakage of urine under stress conditions. For severe SUI, several surgical treatments are available including periurethral bulking injections and suburethral slings, although they present postoperative complications [1, 2]. Cell-based therapy has become an attractive method for treating SUI because it can restore impaired or dysfunctional tissues via the direct effects of transplanted cells [3]. Sources of cells for these therapies include muscle-derived stem cells (MDSCs), bone marrow mesenchymal stem cells, and adipose-derived stem cells (ADSCs). These cells exert therapeutic effects mainly via secretion of a variety of bioactive factors with tissue regenerative, anti-inflammatory, pro-angiogenic, anti-apoptotic, and immunomodulatory properties [4]. Many studies showed that local injection of these cells ameliorated the urethral sphincter dysfunction in animal models of SUI [5]. Furthermore, several clinical trials have shown that local injections of MDSCs and ADSCs are feasible and safe in human SUI, although their efficacy remains uncertain [4, 6].

Dedifferentiated fat (DFAT) cells are ADSC-like multipotent cells that are generated from mature adipocytes by an in vitro dedifferentiation strategy known as ceiling culture [7]. Compared to ADSCs, DFAT cells consist of a higher homogeneous cell population and can be expanded from a smaller amount of adipose tissue regardless of the donor’s age [7, 8]. These characteristic features indicate that the cells are expected to be applicable to autologous cell-based therapy for a variety of diseases including SUI. Our research group reported that local transplantation of DFAT cells contributed to urethral sphincter regeneration with an increased leak point pressure (LPP) in a rat vaginal distension (VD)-induced SUI model [9]. As the limitation of this study, the therapeutic effect could be evaluated only over a short time such as 2 weeks after cell transplantation because the VD model had short durability and recovered to normal voiding function within 4 weeks.

In the present study, we developed a persistent SUI model that allows long-term evaluation using a combination of VD and bilateral ovariectomy (OVX) in rats. In addition, we examined the effect of DFAT cell transplantation on this long-term SUI model in terms of urethral sphincter regeneration.

## Materials and methods

### Experimental animals

Eight-week-old female Sprague–Dawley (SD) rats weighing 200 g were purchased from CLEA Japan, Inc. (Tokyo, Japan). All animal experiments were approved by the Animal Experiment Committee of the Nihon University School of Medicine (AP10M071-4). All experimental procedures were carried out according to the Guide for the Care and Use of Laboratory Animals: Eighth Edition, Washington, DC: The National Academies Press, 2011.

### Cell isolation and culture

Rat DFAT cells were prepared according to the method described previously [10]. Briefly, approximately 1 g of subcutaneous fat tissue was collected, cut into small pieces, and digested in 0.1% (w/v) collagenase type II solution (Sigma-Aldrich, St. Louis, MO, USA) at 37 °C for 30 min with gentle agitation. After filtration and centrifugation at 135×*g* for 3 min, the floating mature adipocyte fraction was collected. After a wash with phosphate-buffered saline (PBS), 5 × 10^4^ adipocytes were inoculated into a 12.5-cm^2^ culture flask (BD Falcon, Franklin Lakes, NJ, USA) filled with Dulbecco’s modified Eagle’s medium (DMEM; Invitrogen, Carlsbad, CA, USA) supplemented with 20% fetal bovine serum (FBS; Lot 627075, Thermo Fisher Scientific, Waltham, MA, USA). The flasks were inverted immediately prior to incubation, and the cells of interest were incubated at the top inner surface (ceiling) of the flasks. At 7 days of culture, the medium was changed to 10%FBS/DMEM, and the flasks were once again inverted, so that the DFAT cells were located at the bottom (floor) of the flasks. The cells were cultured for 7 days and then trypsinized and subcultured on 100-mm tissue culture dishes. The cells were used for experiments within five passages.

Rat fibroblasts were collected and expanded according to the method described previously [11]. The cells were also used for experiments within five passages.

### Rat SUI models

In total, 48 rats were divided into four groups (*n* = 12 in each group): VD group, OVX group, VD + OVX group, and control group. For the VD group, VD was created according to the previously described method [12]. Briefly, under isoflurane inhalation anesthesia, a trimmed 10-Fr Foley catheter was inserted into the rat vagina, and the balloon was inflated with 3 mL of distilled water for 3 h. For the OVX group, bilateral OVX was carried out as described previously [13]. Briefly, under inhalation anesthesia, a small incision was made in the lower and median part of the abdomen, and the bilateral ovaries were identified and removed after ligation on the proximal and distal sides with 3-0 Vicryl. For the VD + OVX group, VD was created under inhalation anesthesia, then OVX was performed 3 h after the VD. For the control group, a sham operation was performed by skin incision only in the abdomen. The LPP was measured in each group at 2, 4, and 6 weeks after the treatment (*n* = 4 for each period in each group). Subsequently, the urethra was removed for histological evaluation.

### DFAT cell transplantation experiment

In total, 14 rats underwent VD and bilateral OVX. Subsequently, 1 × 10^6^ DFAT cells in 20 µm saline (DFAT group, *n* = 7) or 1 × 10^6^ fibroblasts in 20 µm saline (Fibroblast group, *n* = 7) were injected with a Hamilton microsyringe (Hamilton, Reno, NV, USA) into the paraurethral connective tissue at the mid-urethra, which was located at the level of the pubis symphysis. LPP was measured under inhalation anesthesia at 6 weeks after the administration. After measurement, the urethra was harvested, embedded in paraffin, and sectioned (5 µm). The samples were analyzed by immunohistochemistry as described in the histological evaluation section.

### Measurement of LPP

LPP testing was performed with modification of the method reported by Cannon and Damaser [14]. Briefly, rats anesthetized with isoflurane underwent laparotomy to expose the bladder. A polyethylene catheter (0.95 mm in diameter, Becton Dickson, Bedford, MA, USA) was carefully inserted into the bladder dome and fixed. Two days later, rats were anesthetized with urethane (1.0 g/kg), and the catheter was connected to a saline infusion pump and the pressure measuring device by T-branch pipe. The bladder of the rat was made empty manually, and then filled with physiological saline by the infusion pump to determine the maximum bladder volume. After empty the bladder, physiological saline was infused into the bladder at a rate of 5 mL/h to 50% of the maximum bladder volume to avoid overflow. Changes in intravesical pressure during slow and gentle finger pressure on the lower abdomen were recorded until the outflow of urine occurred. From the pressure waveforms, the maximum pressure that caused outflow of urine was regarded as the LPP. The LPP was measured at least 10 times on each rat, and the mean values of the measurements are presented.

### Histological evaluation

The urethra was harvested and fixed in 10% formalin, embedded in paraffin, and sectioned (5 µm) at the level of the mid-urethra. The sections were then deparaffinized and incubated with mouse monoclonal anti-α smooth muscle actin (ASMA) antibody (1:500 dilution, DakoCytomation, Glostrup, Denmark) or mouse monoclonal anti-sarcomeric actin antibody (1:500 dilution, DakoCytomation) overnight. After washing three times with PBS, the samples were incubated with Alexa 594-labeled goat anti-mouse IgG antibody (1:500 dilution, Invitrogen, Carlsbad, CA, USA), or Alexa 488-labeled goat anti-mouse IgG antibody (1:500 dilution, Invitrogen) for 1 h at room temperature. After washing with PBS, nuclei were stained with 5 mg/mL Hoechst 33342 for 30 min at room temperature. Staining was visualized and photographed with a fluorescence microscope (Nikon Eclipse TE 2000-U; Nikon, Tokyo, Japan). For the quantitative analysis, sections of the mid-urethra at the level of the external striated muscle layer with maximum transverse diameter were selected in each sample. Photomicrographs of 5 fields per section chosen at random were taken at 200 × magnification. The mean ASMA-positive area (× 10^–4^ µm^2^) and the mean striated muscle diameter (µm) were measured using a computerized digital morphometric analysis system (Adobe Photoshop CS3 extended, Adobe, San Jose, CA, USA).

### Statistical analysis

The results of LPP measurements and histological quantitative evaluations are expressed as mean value ± standard deviation (SD). One-way analysis of variance (ANOVA) and the Tukey multiple comparison test were used for comparisons between multiple groups. The Mann–Whitney *U* test was used for comparisons between the two groups. *P* < 0.05 was regarded as statistically significant. The statistical analysis was performed with GraphPad Prism ver. 5.0 (GraphPad Software, La Jolla, CA, USA).

## Results

### Analysis of sphincter function and histology in different SUI models

**Fig. 1:**
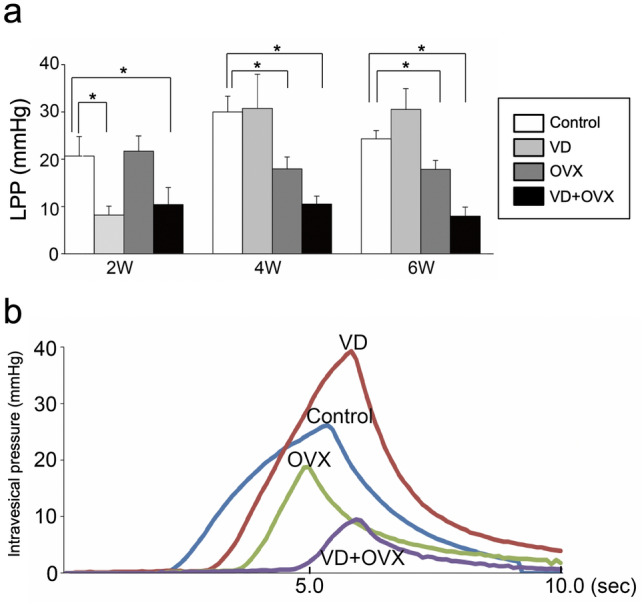
Quantitative evaluation of leak point pressure (LPP) in the different types of stress urinary incontinence (SUI) models. Sprague–Dawley (SD) rats underwent vaginal distention (VD group), bilateral ovariectomy (OVX group), combination of VD and bilateral OVX (VD + OVX group), or sham operation (control group) (*n* = 12 in each group). The LPP was determined at 2, 4, and 6 weeks after treatment. **a** Time-course changes in LPP values in each group after treatment. Data represent mean ± SD. *p < 0.05. **b** Representative LPP waveforms in each group at 6 weeks

We first compared LPP in SD rats receiving VD alone, OVX alone, or the combination of VD and OVX. Time-course changes in LPP in each group are shown in Fig. 1a. In the VD group, LPP was significantly (*p* < 0.05) decreased compared to the control group at 2 weeks after the treatment. The LPP was subsequently recovered at 4 and 6 weeks after the treatment. In the OVX group, there was no particular decrease in LPP at 2 weeks after the treatment, whereas the LPP at 4 and 6 weeks was significantly (*p* < 0.05) decreased compared to that in the control group. In the VD + OVX group, LPP at 2 weeks after the treatment was significantly (*p* < 0.05) decreased compared to that in the control group, similar to that in the VD group. The decreased LPP level was sustained at 4 and 6 weeks. There were significant differences (*p* < 0.05) in the LPP between the control group and the VD + OVX group at 4 and 6 weeks as well as at 2 weeks after the treatment. Representative LPP waveforms in each group at 6 weeks after the treatment are shown in Fig. 1b.

**Fig. 2:**
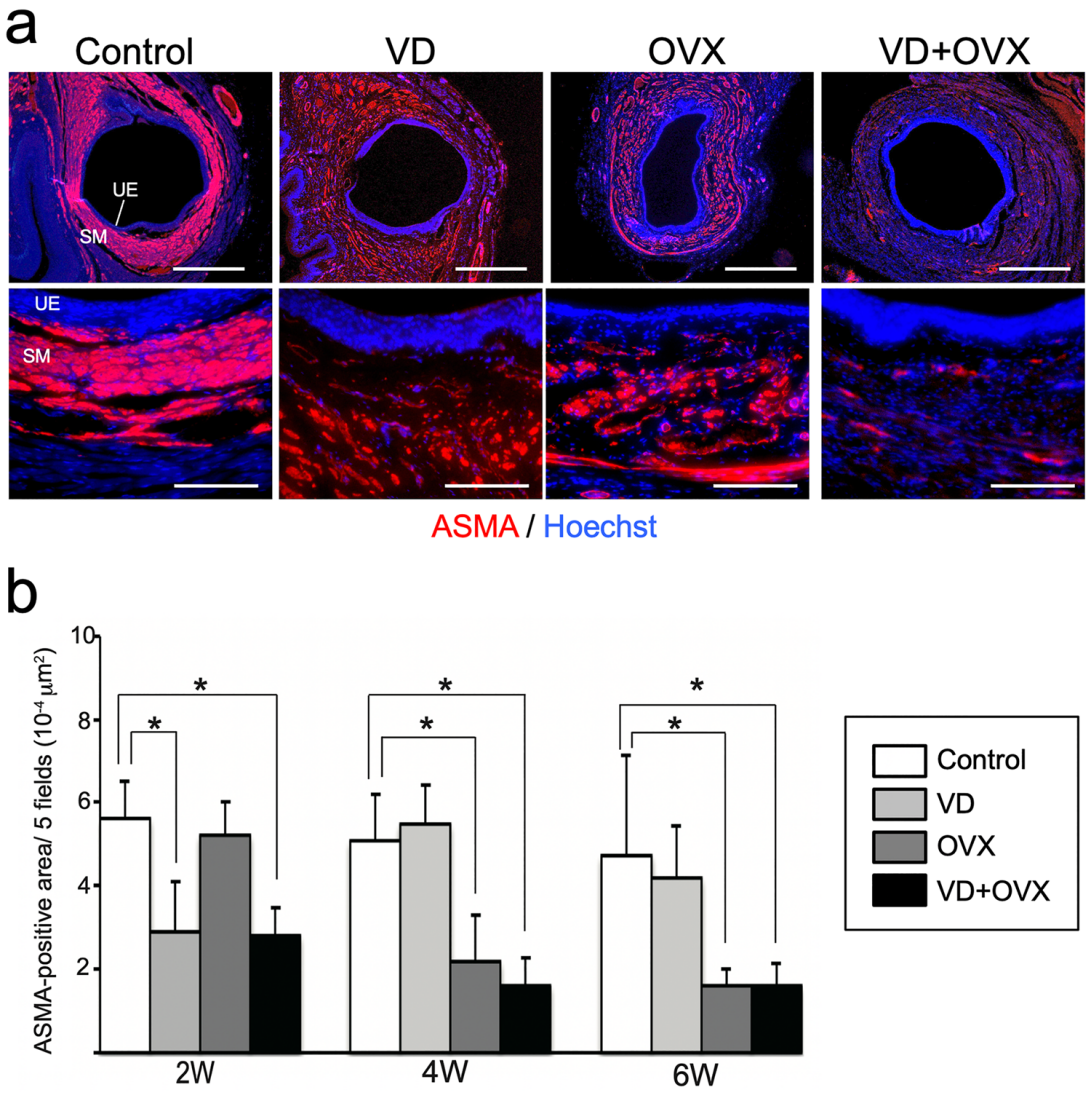
Histological changes of urethral smooth muscle layer in the different types of SUI models. Urethral tissue in each group was sectioned and immunostained for α-smooth muscle actin (ASMA) to detect the internal smooth muscle layer. **a** Representative photomicrographs in each group at 6 weeks after treatment. Lower panels indicate higher-magnification views of the upper panels. Scale bars indicate 500 µm in the upper panels and 100 µm in the lower panels. *UE* urethral epithelium, *SM* smooth muscle layer. **b** Time-course changes of ASMA-positive area in each group after treatment. Data represent mean ± SD. *p < 0.05

**Fig. 3:**
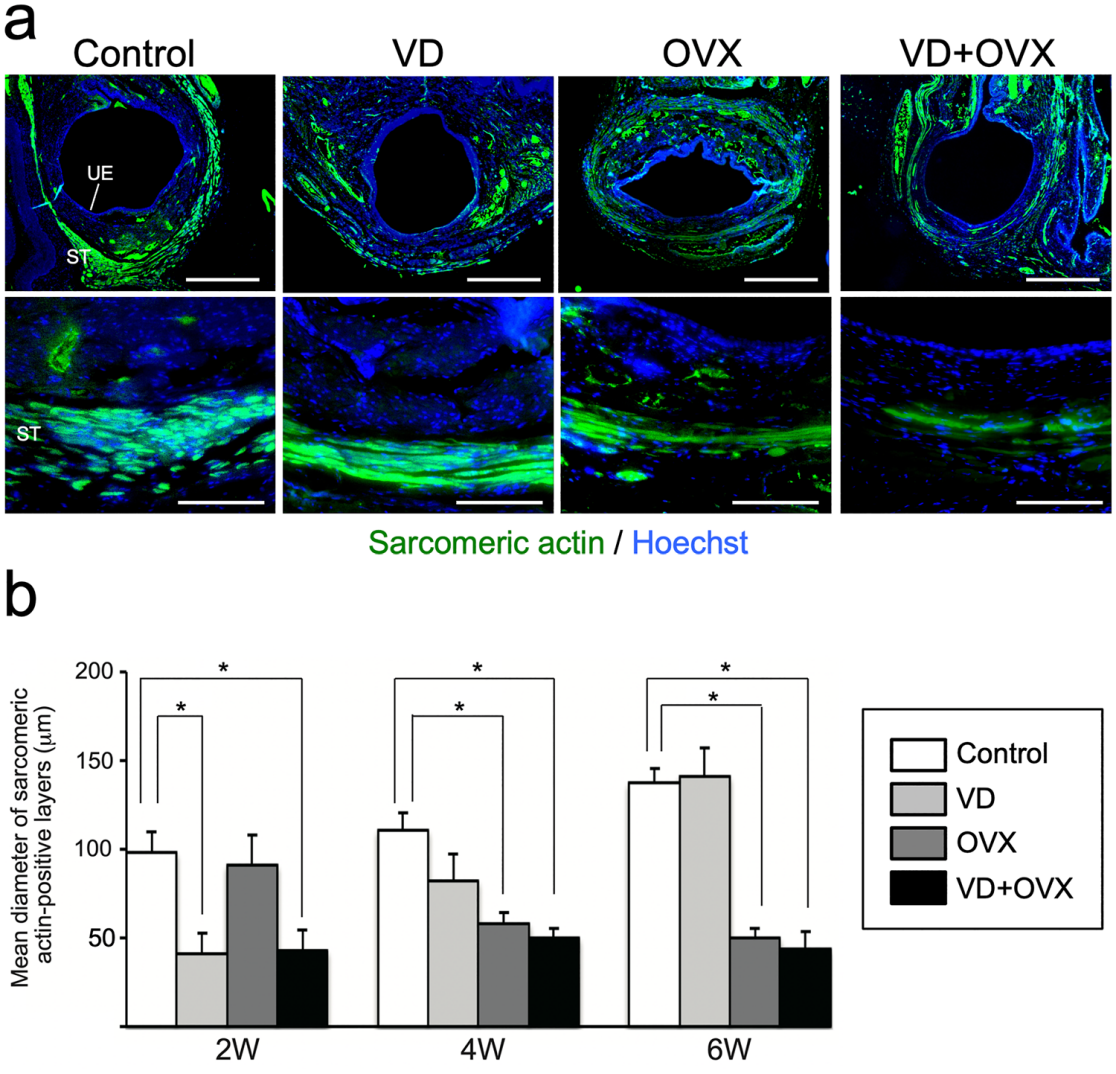
Histological changes of urethral striated muscle layer in the different types of SUI models. Urethral tissue in each group was sectioned and immunostained for sarcomeric actin to detect external striated muscle layer. **a** Representative photomicrographs in each group at 6 weeks after treatment. Lower panels indicate higher-magnification views of the upper panels. Scale bars indicate 500 µm in the upper panel and 100 µm in the lower panel. *UE* urethral epithelium, *ST* striated muscle layer. **b** Time-course changes of mean diameter of sarcomeric actin-positive layers in each group after treatment. Data represent mean ± SD. *p < 0.05

We next evaluated morphological changes of the urethral sphincter in each group by immunohistochemistry. Immunostaining for ASMA at 6 weeks after treatment revealed a prominent atrophic change of the smooth muscle layer with marked fragmentation in the VD + OVX group (Fig. 2a). The time-course changes in the ASMA-positive area in each group are shown in Fig. 2b. The ASMA-positive area in the VD group and VD + OVX group was significantly (*p* < 0.05) decreased at 2 weeks compared to that in the control group. The area in the VD group was subsequently recovered at 4 and 6 weeks, whereas the decreased area in the VD + OVX group remained at 6 weeks. In the OVX group, the area was not altered at 2 weeks but then significantly (*p* < 0.05) decreased at 4 and 6 weeks compared to that in the control group. Immunostaining for sarcomeric actin also revealed the atrophy and thinning of striated muscle layers in the VD + OVX group (Fig. 3a). Time-course changes in the diameter of the striated muscle layer in each group are shown in Fig. 3b. The diameter in the VD group and the VD + OVX group at 2 weeks was significantly (*p* < 0.05) smaller than that in the control group. The decreased diameter in the VD group was gradually recovered at 4 and 6 weeks, whereas that in the VD + OVX group remained at 6 weeks. In the OVX group, the diameter was maintained at 2 weeks, but then significantly (*p* < 0.05) decreased at 4 and 6 weeks compared to that in the control group. These findings indicate that the combination treatment of VD and bilateral OVX induced persistent SUI that exhibited a sustained decrease in LPP with atrophic change of the urethral sphincter at least until 6 weeks after the treatment.

### DFAT cell transplantation experiments in the VD + OVX SUI model

**Fig. 4:**
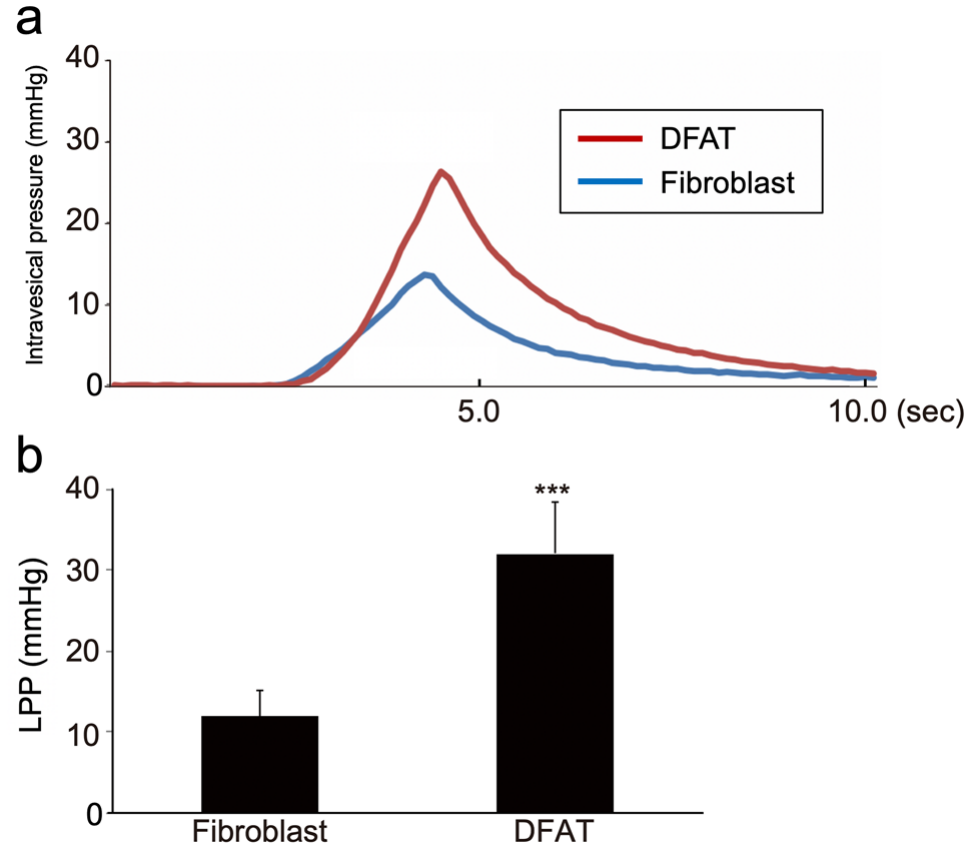
Effect of DFAT cell transplantation on LPP in the persistence SUI model. After combination treatment with VD and bilateral OVX, rat fibroblasts (1 × 10^6^/20 µL, fibroblast group, *n* = 7) or rat DFAT cells (1 × 10^6^/20 µL, DFAT group, *n* = 7) were injected into the urethral tissue, and then the LPP was evaluated 6 weeks after cell transplantation. **a** Representative LPP waveforms in each group. **b** Quantitative evaluation of LPP values in each group. Data represent mean ± SD. ***p < 0.005 vs fibroblast group

**Fig. 5:**
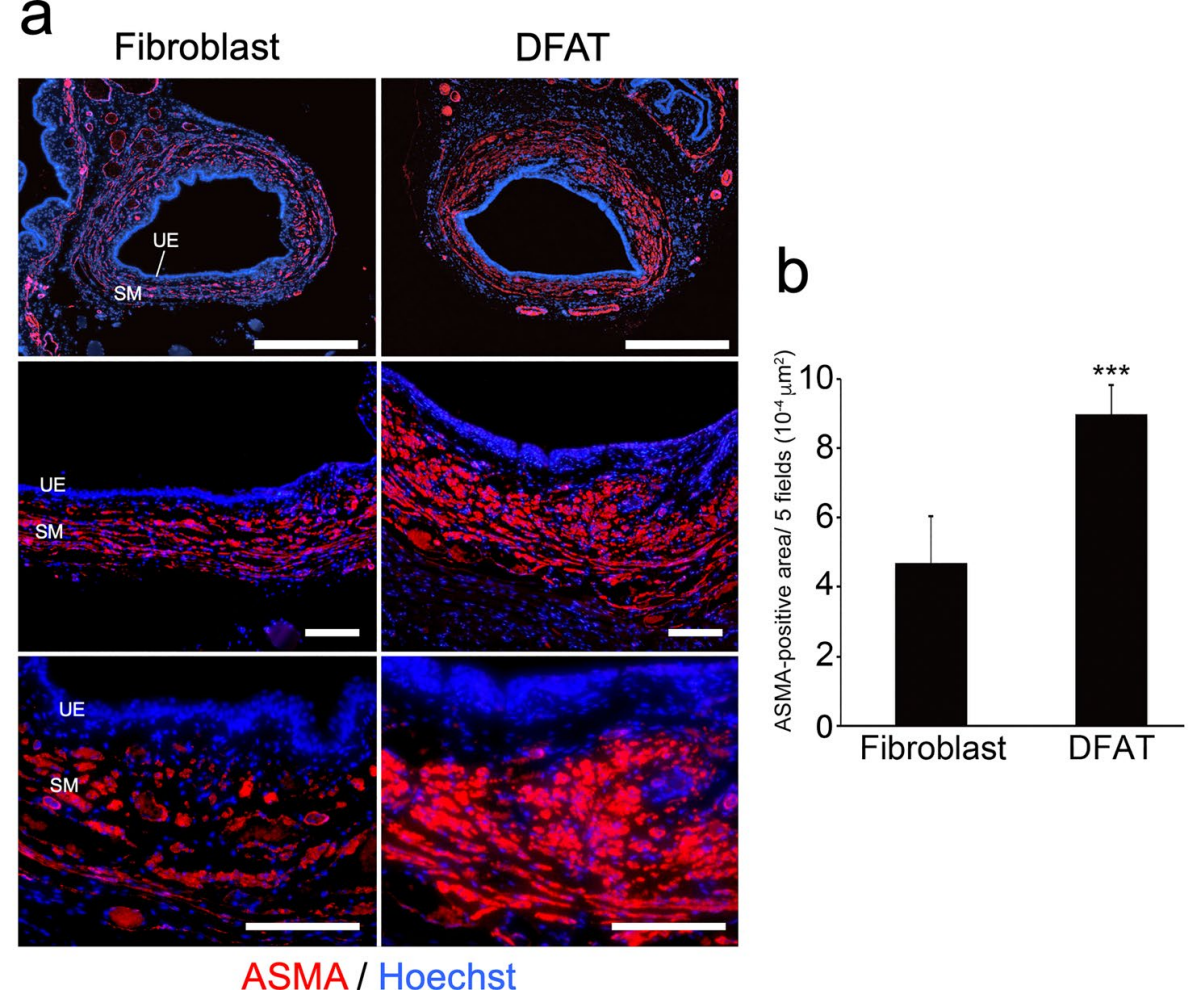
Effect of DFAT cell transplantation on urethral smooth muscle in the persistence SUI model. Urethral tissue in each group was removed at 6 weeks after cell transplantation and immunostained for ASMA to detect internal smooth muscle layer. **a** Representative photomicrographs in each group at 6 weeks after cell transplantation. Middle and lower panels indicate higher-magnification views of the upper panels. Scale bars indicate 500 µm in the upper panels, 100 µm in the middle and lower panels. *UE* urethral epithelium, *SM* smooth muscle layer. **b** Quantitative evaluation of ASMA-positive area in each group. Data represent mean ± SD. ***p < 0.005 vs fibroblast group

**Fig. 6:**
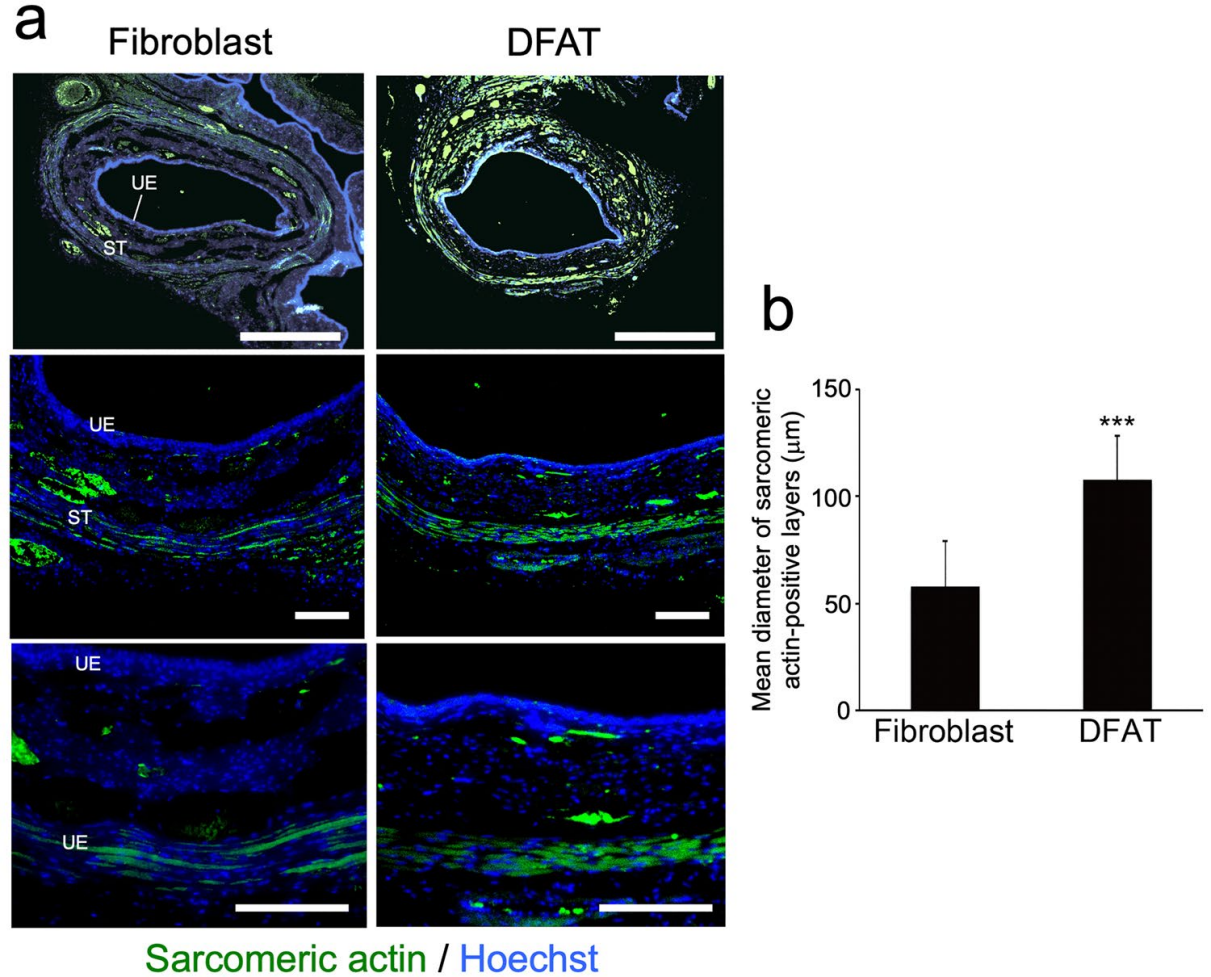
Effect of DFAT cell transplantation on urethral striated muscle in the persistence SUI model. Urethral tissue in each group was removed at 6 weeks after cell transplantation and immunostained for sarcomeric actin to detect the external striated muscle layer. **a** Representative photomicrographs in each group at 6 weeks after cell transplantation. Middle and lower panels indicate higher-magnification views of the upper panels. Scale bars indicate 500 µm in the upper panels, 100 µm in the middle and lower panels. *UE* urethral epithelium, *ST* striated muscle layer. **b** Quantitative evaluation of mean diameter of sarcomeric actin-positive layers in each group. Data represent mean ± SD. ***p < 0.005 vs fibroblast group

To explore the therapeutic potential of DFAT cells for SUI, we next evaluated the effects of DFAT cell transplantation in the persistent SUI model induced by VD and bilateral OVX. Rat dermal fibroblasts were used as a cellular control. Representative LPP waveforms and quantitative data in each group at 6 weeks after treatment are shown in Fig. 4a, b, respectively. LPP in the DFAT group (32.0 ± 6.4 mmHg) was significantly (*p* < 0.005) higher compared to that in the fibroblast group (11.9 ± 3.3 mmHg). Immunostaining for ASMA revealed that the atrophy and fragmentation of the smooth muscle layer in the DFAT group was milder compared to those in the fibroblast group (Fig. 5a). Quantitative analysis confirmed that the ASMA-positive area in the DFAT group (9.0 ± 0.8 × 10^–4^ µm^2^) was significantly (*p* < 0.005) higher than that in the fibroblast group (4.7 ± 1.3 × 10^–4^ µm^2^) (Fig. 5b). Immunostaining for sarcomeric actin revealed thinning of the urethral striated muscle layer in the DFAT group to be milder than that in the fibroblast group (Fig. 6a). The diameter of urethral striated muscle layer in the DFAT group (107.6 ± 20.7 µm) was significantly (*p* < 0.005) higher than that in the fibroblast group (57.8 ± 21.1 µm) (Fig. 6b). These findings indicate that DFAT cell transplantation inhibited urethral muscle degeneration and improved LPP in the persistent SUI model.

## Discussion

The purpose of the present study was twofold: first, to determine a suitable SUI model that allows long-term evaluation of the therapeutic effects of cell transplantation, and second, to examine the effect of DFAT cell transplantation on impaired voiding function using the developed persistent SUI model. Although the VD model is widely used to study SUI associated with childbirth, the model has short durability of about 10 days and recovers to normal voiding function within 6 weeks [15]. To support these previous findings, our previous study showed that the VD-induced decrease in LPP completely recovered by 4 weeks after the treatment [9]. VD is thought to induce ischemic damage and stretch injury to urethral tissue that leads to transient voiding dysfunction. In the present study, we showed that the combination treatment of VD and bilateral OVX induced a sustained decrease in LPP and degradation of the urethral sphincter at least until 6 weeks after the treatment. Similar decreases in LPP by the combination treatment of VD and OVX have been shown in previous studies using mice [16] and rats [17]. Resplande et al. [18] reported that an increased number of apoptotic cells in the urethral epithelium and submucosa were observed even at 9 months after the combination treatment of VD and OVX. Clinical studies revealed that a history of vaginal childbirth and menopause is involved in the incidence of SUI in women [19, 20], and estrogen deficiency is thought to be an essential factor contributing to the pathophysiology of SUI. Cheng and de Groat [21] reported that OVX induced a decrease in bladder–sphincter coordination in rats, and this was recovered by estrogen replacement. Kitta et al. [13] also reported that OVX-induced urethral dysfunction was diminished by estrogen replacement. These findings combined with the present data suggest that OVX-induced estrogen deficiency has an important role in impaired tissue repair after VD, although the mechanism has yet to be elucidated. Our data confirmed that the SUI rat model induced by VD and OVX is suitable for long-term evaluation of cell-based therapy.

In the present study, we showed that periurethral injection of DFAT cells inhibited sphincter muscle atrophy and improved LPP in the persistent SUI model induced by VD and OVX. This study revealed for the first time, to our knowledge, the long-term (6 weeks) therapeutic effects of DFAT cell transplantation for SUI in rats, even though our previous study reported only short-term (2 weeks) beneficial effects of DFAT cell transplantation [9]. Our results showed that injection of DFAT cells was more effective than injection of fibroblasts as a cellular control. It has been reported that periurethral injection of fibroblasts increased the LPP in a bilateral sciatic nerve transection-induced SUI model [22]. However, the effect is thought to be due to a transient bulking effect but not tissue regeneration [15]. Therefore, our results strongly suggest that DFAT cell injection induced morphological and functional regeneration of the urethral sphincter in addition to a bulking effect. Obinata et al. [9] reported that DFAT cell injection promoted the regeneration of urethral sphincter with increased macrophage accumulation. In comparison with fibroblasts, DFAT cells predominantly secrete soluble factors that stimulate monocyte/macrophage migration such as monocyte chemotactic protein-1, macrophage inflammatory protein-3a, and vascular endothelial growth factor. They also reported that injected DFAT cells partially expressed ASMA, suggesting conversion into a myofibroblast/smooth muscle cell phenotype. In vitro study revealed that the differentiation capacity of DFAT cells into myofibroblasts was greater than that of fibroblasts. Thus, it is thought that DFAT cell injection contributes to sphincter muscle regeneration by stimulating macrophage accumulation and direct conversion into a myofibroblast-like phenotype, similar to the phenomenon seen in the wound healing process. Interestingly, a recent study showed that dedifferentiated adipocytes, which were induced by stimulation of skin injury, were involved in skin regeneration via direct myofibroblast conversion and adipocyte lipolysis-induced macrophage accumulation [23].

Adipose tissue-derived cells have gained much attention as attractive cell sources for cell-based therapy because they can be easily and safety harvested in large numbers with minimal morbidity. Several clinical trials using adipose tissue-derived stromal vascular fraction cells [24, 25] and cultured ADSCs [26] to treat SUI were performed and showed long-term safety and efficacy. However, these cells are a heterogenous cell population, and their biological activities are affected by the donor’s age and underlying conditions [27–29]. Mature adipocytes are abundantly present in adipose tissue, and it is feasible to stably collect them from people of any age. After collagenase digestion, mature adipocytes can be easily isolated with high purity by using their buoyancy. Therefore, DFAT cells can be obtained from a smaller amount of adipose tissue (less than 1 g) as a highly homogeneous population compared to stromal vascular fraction cells and cultured ADSCs [7, 8]. In addition, we found that the proliferative and multilineage differentiation abilities of DFAT cells are not affected by donor age and underlying diseases [7]. These properties suggests that DFAT cells are suited for autologous cell-based therapy in patients with SUI that mainly occurs in elderly individuals. Further studies are needed to clarify safety and efficacy in the clinical setting.

Cell-based therapy for SUI has been developed for more than 20 years, however, it is still at the stage of clinical study, not routinely used in daily practice. The current problems of cell therapy for the treatment of SUI include relatively low clinical effectiveness and existence of non-responder patients [4]. To improve the clinical effectiveness, cell type for transplantation, administration dose, and delivery route should be optimized using appropriate animal models of SUI. In addition, therapeutic strategy to focus on not only urethral sphincter reconstruction but also peripheral nerve regeneration should be considered, since reduced nerve density of urethral tissue play an essential role in the development of SUI. Matsumine et al. [30] reported that DFAT cell transplantation contributed to peripheral nerve regeneration in rats, suggesting the potential application of DFAT cells for the treatment of intractable SUI with nerve degeneration.

The present study has certain limitations. First, it is still unclear whether LPP improvement after DFAT cell injection is just due to a bulking effects or additional effects on urethral contractility. Further assessments of sphincteric function such as dynamic urethral pressure recording and urethral sphincter electromyography is needed. Second, we have not confirmed whether injected DFAT cells survive and differentiate into other types of cells up to 6 weeks. Although our previous study showed that DFAT cells survived and partially differentiated into smooth muscle cell phenotype at 2 weeks after transplantation [9], further transplantation experiments using immunofluorescent labeled cells should be conducted to clarify more long-term engrafting.

In conclusion, the combination treatment of VD and bilateral OVX resulted in the creation of a persistent SUI model with atrophic change of the urethral sphincter in rats. In the SUI model, periurethral injection of DFAT cells inhibited sphincter muscle atrophy and improved LPP. The therapeutic effects of DFAT cells were greater than those of fibroblasts. DFAT cell-based therapy may be an attractive therapeutic strategy against SUI.
